# The World Health Organization and global health estimates: improving collaboration and capacity

**DOI:** 10.1186/s12916-015-0286-7

**Published:** 2015-03-10

**Authors:** Ties Boerma, Colin D Mathers

**Affiliations:** Department of Health Statistics and Information Systems, World Health Organization Geneva, 20 Avenue Appia, 1211 Geneva, Switzerland

**Keywords:** Global health, Global health estimates, Health statistics, Population health monitoring, Statistical modelling

## Abstract

Global, regional, and country statistics on population and health indicators are important for assessing development and health progress and for guiding resource allocation; however, data are often lacking, especially in low- and middle-income countries. To fill the gaps, statistical modelling is frequently used to produce comparable health statistics across countries that can be combined to produce regional and global statistics. The World Health Organization (WHO), in collaboration with other United Nations agencies and academic experts, regularly updates estimates for key indicators and involves its Member States in the process. Academic institutions also publish estimates independent from the WHO using different methods. The use of sophisticated statistical estimation methods to fill missing values for countries can reduce the pressures on governments and development agencies to improve information systems. Efforts to improve estimates must be accompanied by concerted attempts to address data gaps, common standards for documentation, sharing of data and methods, and regular interaction and collaboration among all groups involved.

## Introduction: Why does the World Health Organization (WHO) make health estimates?

Global agencies and institutions make extensive use of statistical estimates for key health indicators [1-3]. Estimates are used to track trends over time, make comparisons between populations for particular quantities of interest/indicators, and obtain a comprehensive picture of all causes of death, burden of disease, or risk factors. Agencies publish global and country estimates for a wide range of indicators, in particular mortality levels and trends (life expectancy at different ages, cause-specific mortality (particularly maternal), HIV, tuberculosis, malaria, non-communicable diseases, injuries, etc.). Estimates are also produced for morbidity (mental health, sensory disorders, etc.), coverage of interventions (immunization, skilled birth attendants, etc.), risk factors (hypertension, diabetes, etc.), and financial indicators (total health expenditure).

The estimates have been of considerable value in generating an overview of the health situation and emerging trends and for reporting on country and global progress towards international goals and targets such as the Millennium Development Goals. The production and dissemination of health statistics for health action at the country, regional, and global levels are core WHO activities mandated by the Member States in the Constitution. WHO figures carry great weight in national and international resource allocation, policy-making, and programming given the WHO’s reputation as being unbiased (impartial and fair), global (having a worldwide remit and responsibility), and technically competent (drawing on leading research and policy institutions and individuals). The WHO works closely with countries, partners, and global experts to produce health statistics of the greatest possible accuracy. Periodic updates of global health estimates usually involve statistical modelling to overcome major gaps in country data availability and quality and to obtain comparable global, regional, and country health statistics.

## How the WHO seeks to obtain accurate, internationally-agreed health estimates

There are many options in statistical modelling in terms of type of model, assumptions, and complexity. An important feature of the WHO’s global health monitoring is its commitment to transparency and consensus. This is achieved in several ways. First, the WHO collaborates with other UN agencies to combine technical resources and ensure that a single UN estimate is provided, for instance, for monitoring progress towards the targets for the Millennium Development Goals. Second, technical advisory groups comprised of independent academic experts provide methodological advice to the WHO and other collaborating UN agencies on health estimates. Examples include the Interagency Child mortality Estimation Group, Maternal Mortality Expert Group, Child Health Epidemiology Research Group and Maternal Child Epidemiology Estimation (causes of death in children, secretariat at Johns Hopkins University), multiple disease expert groups (e.g., QUIVER for vaccine-preventable diseases, UN reference groups for malaria and AIDS, IARC and WHO for cancers), and adult mortality collaborations (WHO and UN Population Division). Third, the country consultation process provides a platform for member states to understand how estimates are derived, and for the WHO to identify additional data sources that can be used to improve the accuracy of estimates. It is a consultation process, not a clearance, meaning that WHO and country best estimates may differ because of differences in data and methodology used. Fourth, the WHO and collaborating agencies have set a standard for transparency and reproducibility, as input datasets and statistical code/modelling software are now freely available for several major disease areas (maternal mortality [4], child causes of death [5], infant and child mortality [6], and HIV/AIDS [7]), allowing Member States and other interested parties to understand and replicate those analyses. The goal is to have all estimates accompanied by tools that can be used by countries to better understand the estimates and alter input data and assumptions as appropriate. Fifth, the WHO policy now aims to ensure that all peer-reviewed journal articles with involvement of WHO staff will be published as open access.

## How WHO works with other organizations

Estimates always have uncertainty and the fewer quality data points, the greater the uncertainty. Different researchers can easily come up with different estimates for the same country, region, or globally and this has happened on many occasions. In recent years, the Institute for Health Metrics and Evaluation (IHME) at the University of Washington has started to publish, often in *The Lancet*, estimates for many health indicators globally and for countries. Sometimes, these estimates are very different from those published by the WHO and UN agencies.

For example, the IHME Global Burden of Disease 2010 study estimated that there were 1.24 million deaths due to malaria in 2010, with more than half a million of these occurring in those aged 5 years and older [8]. These estimates were substantially larger than those of the WHO at the time: 655,000 deaths in total, with less than 100,000 in those aged 5 years and over [9]. The most recent IHME estimate is lower, at 855,000 in 2013, but still substantially higher than current WHO estimates [10]. IHME also estimated less than 200,000 child tuberculosis cases in 2013, much less than the approximately 350,000 cases notified to the WHO in 2012, and substantially less than the WHO estimate of 530,000 cases and two other recently published independent estimates [11,12]. IHME estimates of all-cause mortality rates vary substantially in some cases from those of the United Nations Population Division. For example, IHME estimated there were 817,000 deaths for children aged 5 to 14 years in 2010 [13], only 57% of the 1.44 million estimated by United Nations Population Division [14].

The WHO reviews methods and estimates developed by IHME and other organizations, and may make use of them in cases where scientific rigor can be evaluated. For instance, the publication of the IHME Global Burden of Disease 2010 Study [15] in 2012 led to consultations and exchange of data between IHME and the WHO and other UN agencies and their expert groups. Some results from the IHME Global Burden of Disease 2010 study have also been used by the WHO in preparing its global health statistics [16].

## Dealing with different estimates

The great investments in global health estimates, mainly by the Bill and Melinda Gates Foundation into IHME, have led to a sharp increase in the number of estimates. Over the past few years, investigation into differences in estimates for the same indicator has led to improvements in data inputs and estimation methods used by IHME and UN agencies. The existence of divergent estimates for the same indicator has led to increased awareness of major data gaps, especially in low- and middle-income countries. Lack of reliable data implies greater reliance on borrowing data from other – often higher income countries – and covariates to predict country statistics.

Globally, the existence of multiple estimates for the same indicator, albeit often with large overlapping uncertainty ranges has led to some concerns, voiced in international meetings. Countries seem less worried; to date, no country has challenged the WHO draft estimates during the country consultation process on the basis of the existence of a competing estimate.

In several areas there is convergence in terms of methods and results of the estimation modelling. Examples include child mortality, maternal mortality, and etiology of pneumonia. In others, more work is needed to discuss data inputs, methods, and discrepant results.

## Improving global collaboration

The global field of estimates requires further collaboration between IHME, other academic institutions, the WHO, and other UN agencies through expert group mechanisms. In addition to expert group mechanisms that exist for specific programs, the WHO has established an overall reference group on health statistics [17]. The reference group’s agenda for 2014 to 2015 includes the establishment of guidance for reporting global health estimates, the improvement of methods to estimate the overall number of deaths from life tables, the development of a single global standard instrument for verbal autopsy (which is now being field tested), and ways to enhance the use of estimate for country decision making. In addition, WHO headquarters and regional offices are developing a memorandum of understanding with IHME to enhance collaboration. This memorandum is intended to encourage collaboration on country capacity strengthening, data sharing, and interaction on methods, tools, and actual global health estimates.

## Improving country-specific capacity for data collection and analysis

In addition to improving global collaboration, there is an urgent need to enhance country capacity for collection, analysis, and use of health data and statistics. In fact, there are relatively few countries that use global estimates for national decision-making on a regular basis. First, major data gaps, such as the lack of reliable data on deaths by age, sex, and cause in over 100 countries, are still a major obstacle and need to be addressed. Second, country capacity to analyze health data, adjust for biases, reconcile data from different sources, and produce estimates for key indicators needs to be strengthened considerably. This includes multi-year training programs, but also user-friendly analytical tools to examine data and produce estimates. Third, the WHO and partners need to collaborate to enhance country capacity for effective communication and use of health statistics to support key country planning and monitoring processes such as health sector strategic plans and their regular reviews. The WHO is particularly well-positioned with its country offices working closely with ministries of health and local health institutions. Only a persistent joint effort of the WHO, other UN agencies, academic institutions, and others can make a difference and lead to sustained improvements in information systems and data and analytic capacity in countries.

## Conclusions

Global, regional, and country statistics on population and health indicators are important because they help guide health investment priorities, allow assessment of progress and of intervention effectiveness, and are needed for holding national and international health agencies accountable, and for guiding resource allocation. Timely reliable data are often lacking for many indicators, especially in low- and middle-income countries. Because of the major gaps and inconsistencies in existing health information, statistical modelling is frequently used to produce comparable health statistics across countries that can be combined to produce regional and global statistics. In addition, modelling is often needed to provide a comprehensive assessment of causes of death and ill-health.

Academic institutions are increasingly publishing estimates in parallel to the WHO using different methods which may result in substantially different results. *The Lancet* has become a regular channel for publication of global, regional, and country statistics on key health indicators and the burden of disease. Rudan and Chan recently characterized this as a competitive situation which is challenging the position of the WHO [18].

We do not regard this situation as necessarily competitive. The WHO and other UN agencies will continue to prepare and report on global health indicators to fulfil their mandate from Member States, and to be accountable to those States through a transparent process, reproducible methods, and country involvement. Academic inputs are needed to improve data collection, compilation, and sharing, analytical methods, and communication of global health indicators. For many years this has almost exclusively occurred in the context of the WHO or UN expert groups, and now this work is also taking place in independent academic research institutions, most commonly through IHME’s work on the global burden of disease. The resulting debates on data interpretation, methods, and results can be healthy and productive if the debates can ensure that focus on methodological sophistication does not go at the expense of working together to improve the essential investments in data collection, analysis, and use in low- and middle-income countries. Efforts to improve collaboration on estimates are critical and must be accompanied by concerted attempts to address data gaps, particularly for death registration with information on causes of death, and to improve public transparency and data availability.

## Abbreviations

IHME: Institute for Health Metrics and Evaluation
WHO: World Health Organization
